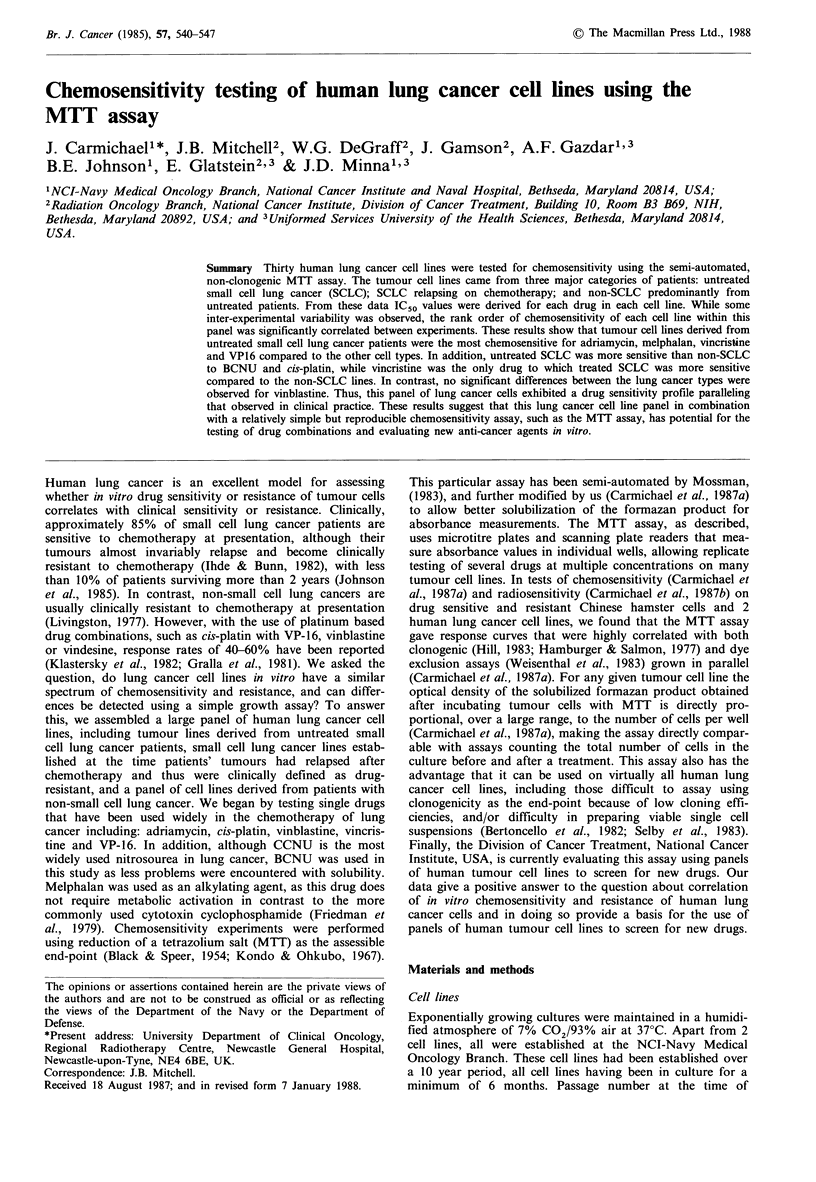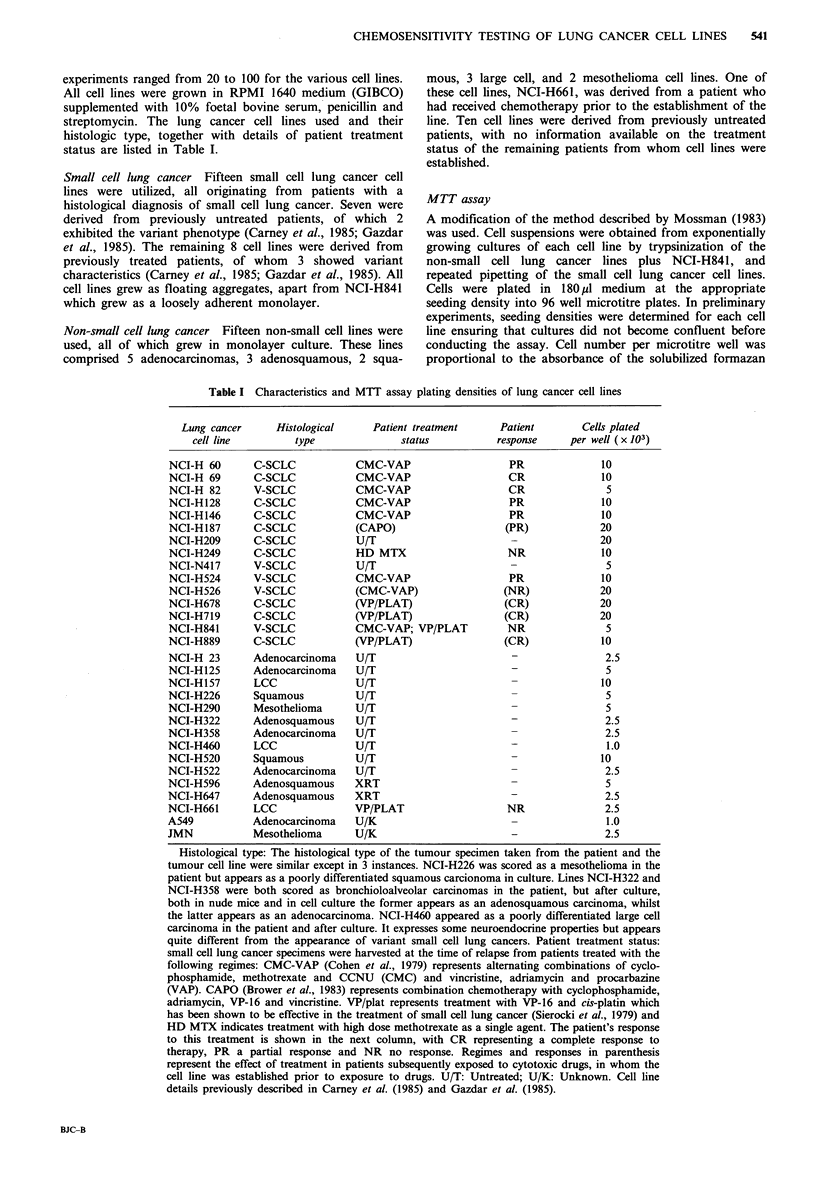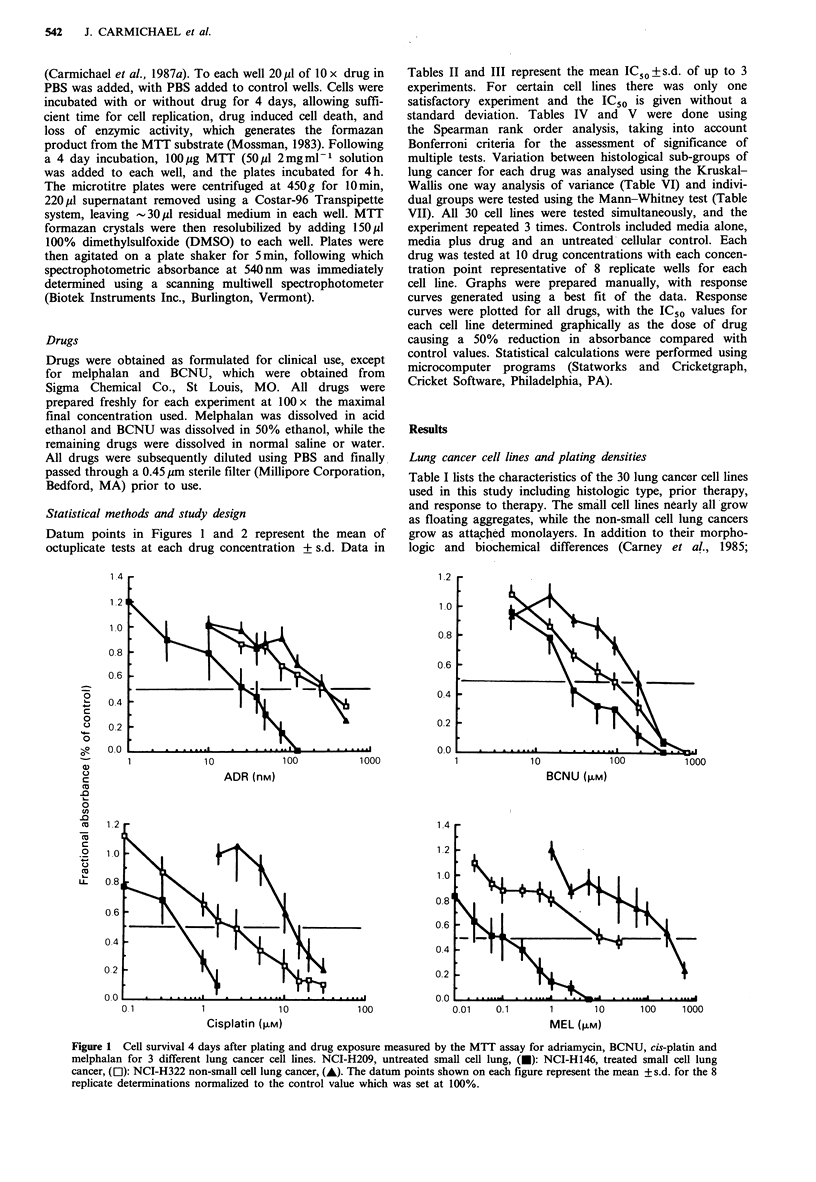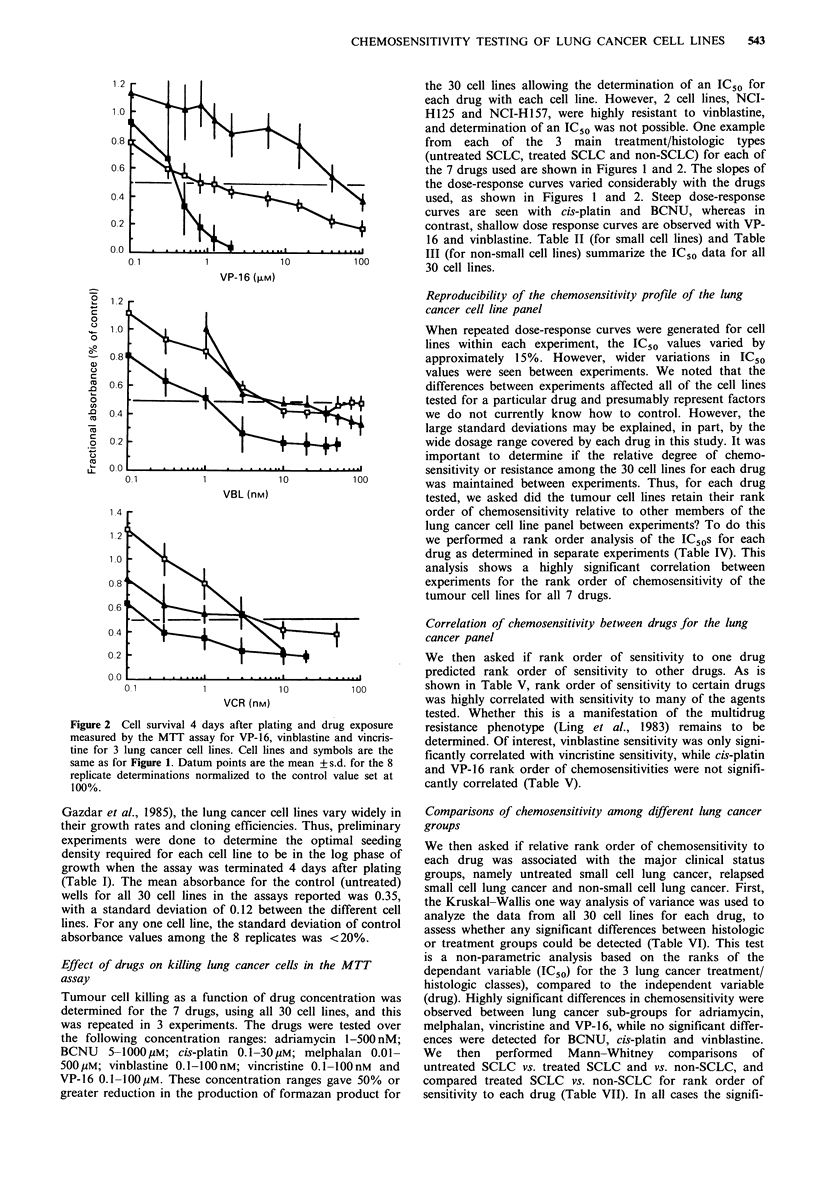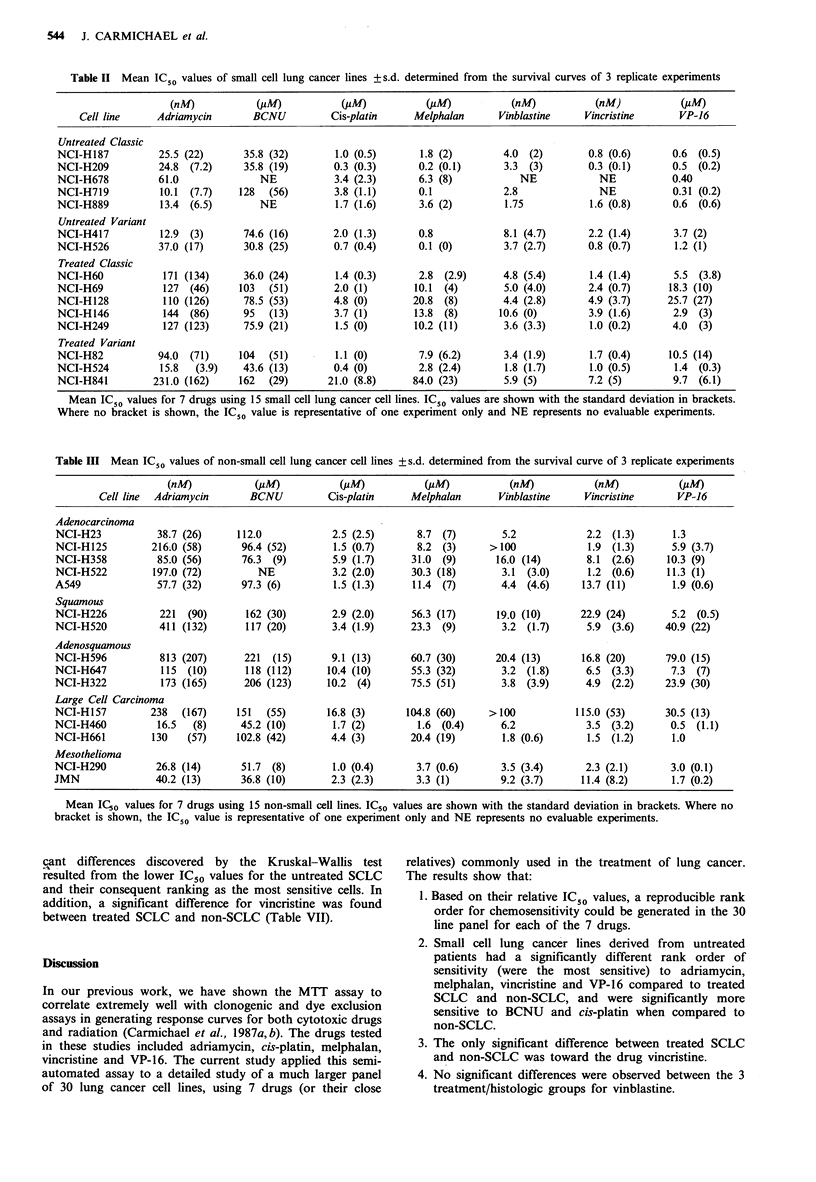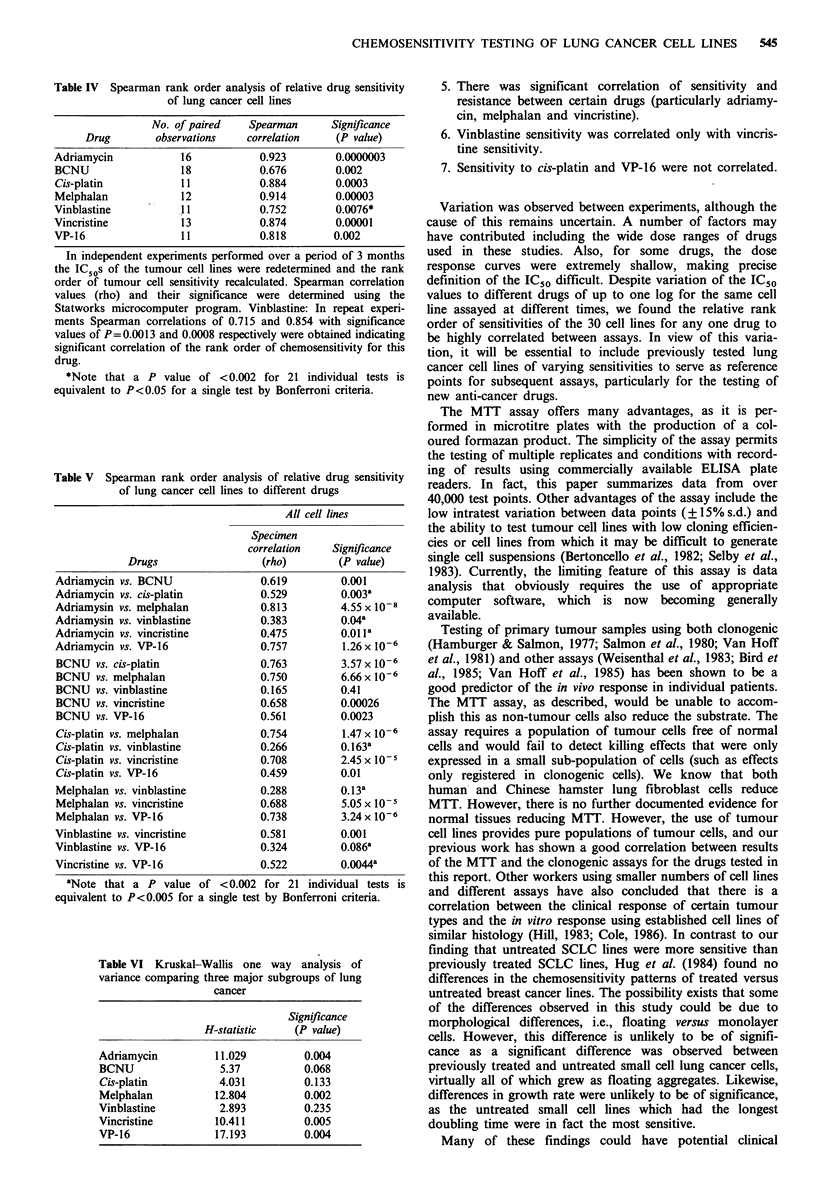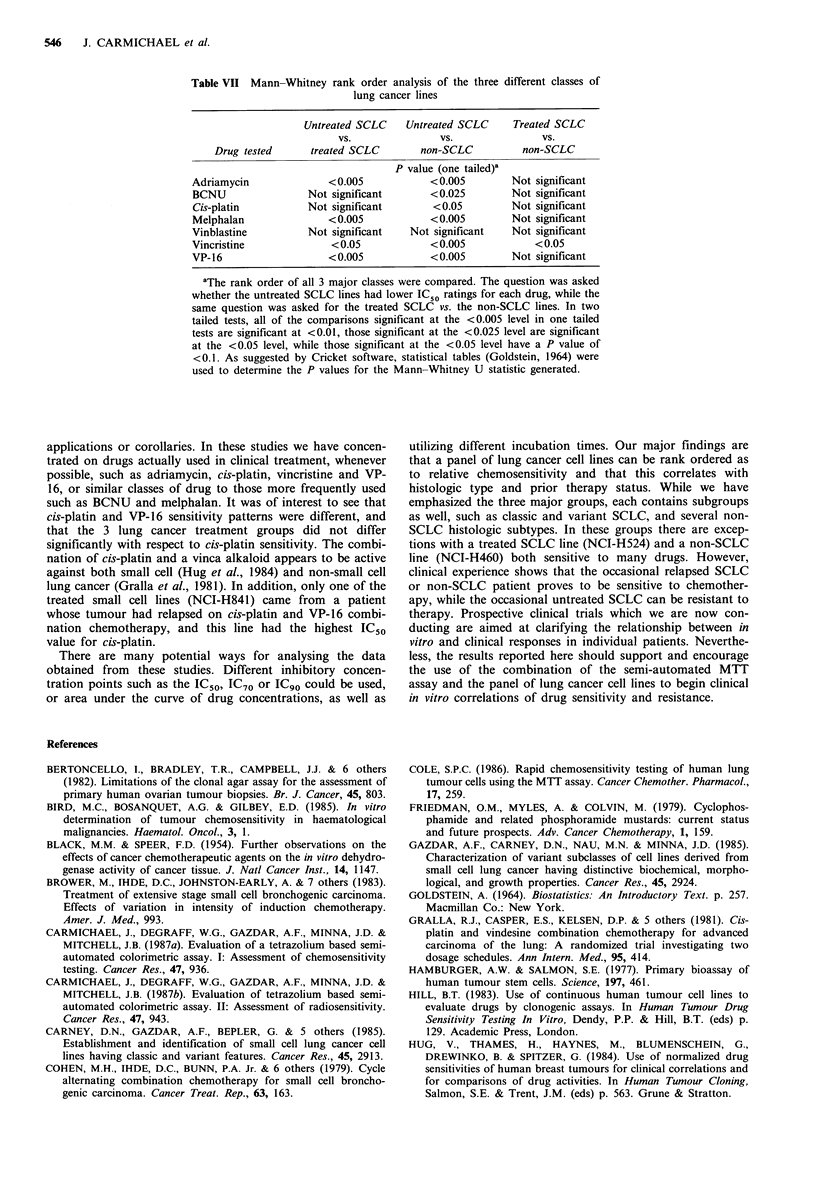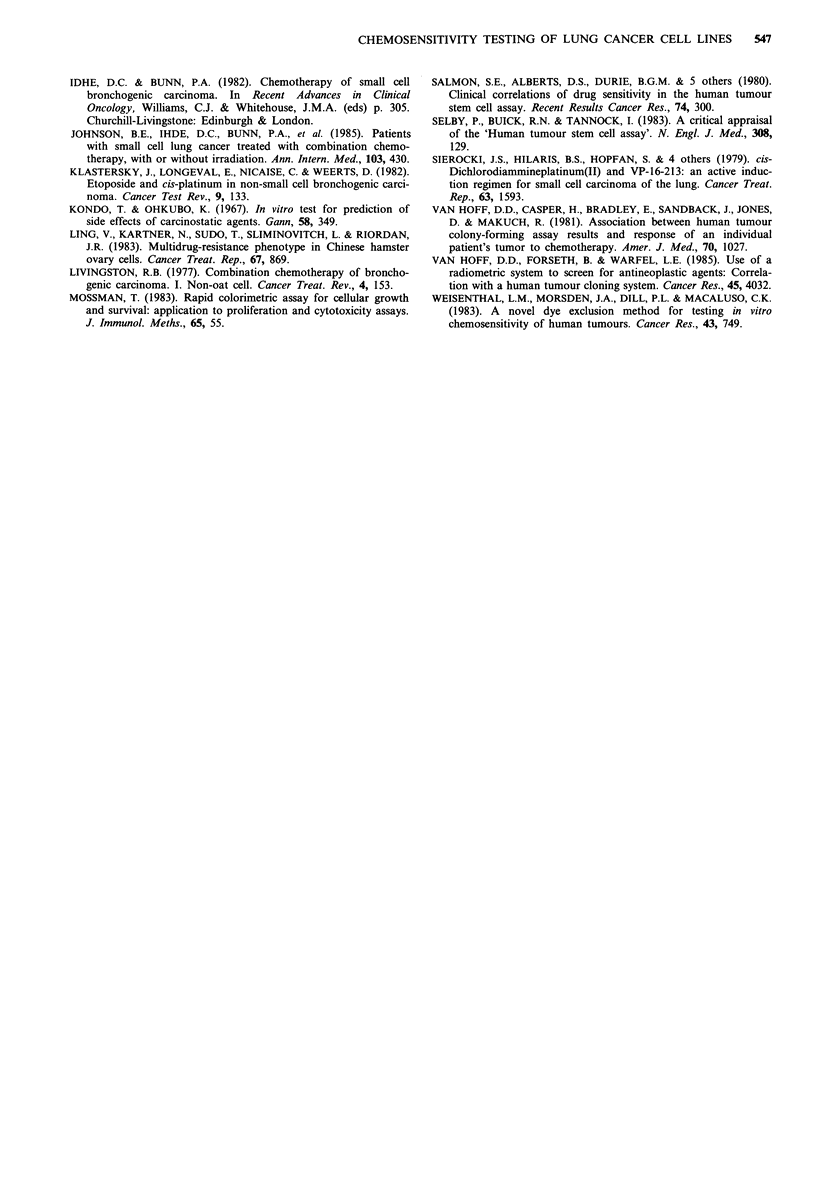# Chemosensitivity testing of human lung cancer cell lines using the MTT assay.

**DOI:** 10.1038/bjc.1988.125

**Published:** 1988-06

**Authors:** J. Carmichael, J. B. Mitchell, W. G. DeGraff, J. Gamson, A. F. Gazdar, B. E. Johnson, E. Glatstein, J. D. Minna

**Affiliations:** NCI-Navy Medical Oncology Branch, Bethesda, Maryland 20814.

## Abstract

Thirty human lung cancer cell lines were tested for chemosensitivity using the semi-automated, non-clonogenic MTT assay. The tumour cell lines came from three major categories of patients: untreated small cell lung cancer (SCLC); SCLC relapsing on chemotherapy; and non-SCLC predominantly from untreated patients. From these data IC50 values were derived for each drug in each cell line. While some inter-experimental variability was observed, the rank order of chemosensitivity of each cell line within this panel was significantly correlated between experiments. These results show that tumour cell lines derived from untreated small cell lung cancer patients were the most chemosensitive for adriamycin, melphalan, vincristine and VP16 compared to the other cell types. In addition, untreated SCLC was more sensitive than non-SCLC to BCNU and cis-platin, while vincristine was the only drug to which treated SCLC was more sensitive compared to the non-SCLC lines. In contrast, no significant differences between the lung cancer types were observed for vinblastine. Thus, this panel of lung cancer cells exhibited a drug sensitivity profile paralleling that observed in clinical practice. These results suggest that this lung cancer cell line panel in combination with a relatively simple but reproducible chemosensitivity assay, such as the MTT assay, has potential for the testing of drug combinations and evaluating new anti-cancer agents in vitro.


Br. J. Cancer (1985), 57, 540–547 © The Macmillan Press Ltd., 1988

### Chemosensitivity testing of human lung cancer cell lines using the MTT assay

J. Carmichael[1]*, J.B. Mitchell[2], W.G. DeGraff[2], J. Gamson[2], A.F. Gazdar[1,3]

B.E. Johnson[1], E. Glatstein[2,3] & J.D. Minna[1,3]

[1]*NCI-Navy Medical Oncology Branch, National Cancer Institute and Naval Hospital, Bethseda, Maryland 20814, USA;* [2]*Radiation Oncology Branch, National Cancer Institute, Division of Cancer Treatment, Building 10, Room B3 B69, NIH, Bethesda, Maryland 20892, USA; and* [3]*Uniformed Services University of the Health Sciences, Bethesda, Maryland 20814, USA.*

**Summary** Thirty human lung cancer cell lines were tested for chemosensitivity using the semi-automated, non-clonogenic MTT assay. The tumour cell lines came from three major categories of patients: untreated small cell lung cancer (SCLC); SCLC relapsing on chemotherapy; and non-SCLC predominantly from untreated patients. From these data $IC_{50}$ values were derived for each drug in each cell line. While some inter-experimental variability was observed, the rank order of chemosensitivity of each cell line within this panel was significantly correlated between experiments. These results show that tumour cell lines derived from untreated small cell lung cancer patients were the most chemosensitive for adriamycin, melphalan, vincristine and VP16 compared to the other cell types. In addition, untreated SCLC was more sensitive than non-SCLC to BCNU and *cis*-platin, while vincristine was the only drug to which treated SCLC was more sensitive compared to the non-SCLC lines. In contrast, no significant differences between the lung cancer types were observed for vinblastine. Thus, this panel of lung cancer cells exhibited a drug sensitivity profile paralleling that observed in clinical practice. These results suggest that this lung cancer cell line panel in combination with a relatively simple but reproducible chemosensitivity assay, such as the MTT assay, has potential for the testing of drug combinations and evaluating new anti-cancer agents *in vitro*.

Human lung cancer is an excellent model for assessing whether *in vitro* drug sensitivity or resistance of tumour cells correlates with clinical sensitivity or resistance. Clinically, approximately 85% of small cell lung cancer patients are sensitive to chemotherapy at presentation, although their tumours almost invariably relapse and become clinically resistant to chemotherapy (Ihde & Bunn, 1982), with less than 10% of patients surviving more than 2 years (Johnson *et al*., 1985). In contrast, non-small cell lung cancers are usually clinically resistant to chemotherapy at presentation (Livingston, 1977). However, with the use of platinum based drug combinations, such as *cis*-platin with VP-16, vinblastine or vindesine, response rates of 40–60% have been reported (Klastersky *et al*., 1982; Gralla *et al*., 1981). We asked the question, do lung cancer cell lines *in vitro* have a similar spectrum of chemosensitivity and resistance, and can differences be detected using a simple growth assay? To answer this, we assembled a large panel of human lung cancer cell lines, including tumour lines derived from untreated small cell lung cancer patients, small cell lung cancer lines established at the time patients' tumours had relapsed after chemotherapy and thus were clinically defined as drug-resistant, and a panel of cell lines derived from patients with non-small cell lung cancer. We began by testing single drugs that have been used widely in the chemotherapy of lung cancer including: adriamycin, *cis*-platin, vinblastine, vincristine and VP-16. In addition, although CCNU is the most widely used nitrosourea in lung cancer, BCNU was used in this study as less problems were encountered with solubility. Melphalan was used as an alkylating agent, as this drug does not require metabolic activation in contrast to the more commonly used cytotoxin cyclophosphamide (Friedman *et al*., 1979). Chemosensitivity experiments were performed using reduction of a tetrazolium salt (MTT) as the assessible end-point (Black & Speer, 1954; Kondo & Ohkubo, 1967). This particular assay has been semi-automated by Mossman, (1983), and further modified by us (Carmichael *et al*., 1987*a*) to allow better solubilization of the formazan product for absorbance measurements. The MTT assay, as described, uses microtitre plates and scanning plate readers that measure absorbance values in individual wells, allowing replicate testing of several drugs at multiple concentrations on many tumour cell lines. In tests of chemosensitivity (Carmichael *et al*., 1987*a*) and radiosensitivity (Carmichael *et al*., 1987*b*) on drug sensitive and resistant Chinese hamster cells and 2 human lung cancer cell lines, we found that the MTT assay gave response curves that were highly correlated with both clonogenic (Hill, 1983; Hamburger & Salmon, 1977) and dye exclusion assays (Weisenthal *et al*., 1983) grown in parallel (Carmichael *et al*., 1987*a*). For any given tumour cell line the optical density of the solubilized formazan product obtained after incubating tumour cells with MTT is directly proportional, over a large range, to the number of cells per well (Carmichael *et al*., 1987*a*), making the assay directly comparable with assays counting the total number of cells in the culture before and after a treatment. This assay also has the advantage that it can be used on virtually all human lung cancer cell lines, including those difficult to assay using clonogenicity as the end-point because of low cloning efficiencies, and/or difficulty in preparing viable single cell suspensions (Bertoncello *et al*., 1982; Selby *et al*., 1983). Finally, the Division of Cancer Treatment, National Cancer Institute, USA, is currently evaluating this assay using panels of human tumour cell lines to screen for new drugs. Our data give a positive answer to the question about correlation of *in vitro* chemosensitivity and resistance of human lung cancer cells and in doing so provide a basis for the use of panels of human tumour cell lines to screen for new drugs.

### Materials and methods

### *Cell lines*

Exponentially growing cultures were maintained in a humidified atmosphere of 7% $CO_2$/93% air at 37°C. Apart from 2 cell lines, all were established at the NCI-Navy Medical Oncology Branch. These cell lines had been established over a 10 year period, all cell lines having been in culture for a minimum of 6 months. Passage number at the time of

The opinions or assertions contained herein are the private views of the authors and are not to be construed as official or as reflecting the views of the Department of the Navy or the Department of Defense.

*Present address: University Department of Clinical Oncology, Regional Radiotherapy Centre, Newcastle General Hospital, Newcastle-upon-Tyne, NE4 6BE, UK.
Correspondence: J.B. Mitchell.

Received 18 August 1987; and in revised form 7 January 1988.

experiments ranged from 20 to 100 for the various cell lines. All cell lines were grown in RPMI 1640 medium (GIBCO) supplemented with 10% foetal bovine serum, penicillin and streptomycin. The lung cancer cell lines used and their histologic type, together with details of patient treatment status are listed in Table I.

*Small cell lung cancer* Fifteen small cell lung cancer cell lines were utilized, all originating from patients with a histological diagnosis of small cell lung cancer. Seven were derived from previously untreated patients, of which 2 exhibited the variant phenotype (Carney *et al.*, 1985; Gazdar *et al.*, 1985). The remaining 8 cell lines were derived from previously treated patients, of whom 3 showed variant characteristics (Carney *et al.*, 1985; Gazdar *et al.*, 1985). All cell lines grew as floating aggregates, apart from NCI-H841 which grew as a loosely adherent monolayer.

*Non-small cell lung cancer* Fifteen non-small cell lines were used, all of which grew in monolayer culture. These lines comprised 5 adenocarcinomas, 3 adenosquamous, 2 squamous, 3 large cell, and 2 mesothelioma cell lines. One of these cell lines, NCI-H661, was derived from a patient who had received chemotherapy prior to the establishment of the line. Ten cell lines were derived from previously untreated patients, with no information available on the treatment status of the remaining patients from whom cell lines were established.

### *MTT assay*

A modification of the method described by Mossman (1983) was used. Cell suspensions were obtained from exponentially growing cultures of each cell line by trypsinization of the non-small cell lung cancer lines plus NCI-H841, and repeated pipetting of the small cell lung cancer cell lines. Cells were plated in 180 $\mu$l medium at the appropriate seeding density into 96 well microtitre plates. In preliminary experiments, seeding densities were determined for each cell line ensuring that cultures did not become confluent before conducting the assay. Cell number per microtitre well was proportional to the absorbance of the solubilized formazan

**Table I** Characteristics and MTT assay plating densities of lung cancer cell lines

| *Lung cancer cell line* | *Histological type* | *Patient treatment status* | *Patient response* | *Cells plated per well* ($\times 10^3$) |
|---|---|---|---|---|
| NCI-H 60 | C-SCLC | CMC-VAP | PR | 10 |
| NCI-H 69 | C-SCLC | CMC-VAP | CR | 10 |
| NCI-H 82 | V-SCLC | CMC-VAP | CR | 5 |
| NCI-H128 | C-SCLC | CMC-VAP | PR | 10 |
| NCI-H146 | C-SCLC | CMC-VAP | PR | 10 |
| NCI-H187 | C-SCLC | (CAPO) | (PR) | 20 |
| NCI-H209 | C-SCLC | U/T | – | 20 |
| NCI-H249 | C-SCLC | HD MTX | NR | 10 |
| NCI-N417 | V-SCLC | U/T | – | 5 |
| NCI-H524 | V-SCLC | CMC-VAP | PR | 10 |
| NCI-H526 | V-SCLC | (CMC-VAP) | (NR) | 20 |
| NCI-H678 | C-SCLC | (VP/PLAT) | (CR) | 20 |
| NCI-H719 | C-SCLC | (VP/PLAT) | (CR) | 20 |
| NCI-H841 | V-SCLC | CMC-VAP; VP/PLAT | NR | 5 |
| NCI-H889 | C-SCLC | (VP/PLAT) | (CR) | 10 |
| NCI-H 23 | Adenocarcinoma | U/T | – | 2.5 |
| NCI-H125 | Adenocarcinoma | U/T | – | 5 |
| NCI-H157 | LCC | U/T | – | 10 |
| NCI-H226 | Squamous | U/T | – | 5 |
| NCI-H290 | Mesothelioma | U/T | – | 5 |
| NCI-H322 | Adenosquamous | U/T | – | 2.5 |
| NCI-H358 | Adenocarcinoma | U/T | – | 2.5 |
| NCI-H460 | LCC | U/T | – | 1.0 |
| NCI-H520 | Squamous | U/T | – | 10 |
| NCI-H522 | Adenocarcinoma | U/T | – | 2.5 |
| NCI-H596 | Adenosquamous | XRT | – | 5 |
| NCI-H647 | Adenosquamous | XRT | – | 2.5 |
| NCI-H661 | LCC | VP/PLAT | NR | 2.5 |
| A549 | Adenocarcinoma | U/K | – | 1.0 |
| JMN | Mesothelioma | U/K | – | 2.5 |

Histological type: The histological type of the tumour specimen taken from the patient and the tumour cell line were similar except in 3 instances. NCI-H226 was scored as a mesothelioma in the patient but appears as a poorly differentiated squamous carcionoma in culture. Lines NCI-H322 and NCI-H358 were both scored as bronchioloalveolar carcinomas in the patient, but after culture, both in nude mice and in cell culture the former appears as an adenosquamous carcinoma, whilst the latter appears as an adenocarcinoma. NCI-H460 appeared as a poorly differentiated large cell carcinoma in the patient and after culture. It expresses some neuroendocrine properties but appears quite different from the appearance of variant small cell lung cancers. Patient treatment status: small cell lung cancer specimens were harvested at the time of relapse from patients treated with the following regimes: CMC-VAP (Cohen *et al.*, 1979) represents alternating combinations of cyclophosphamide, methotrexate and CCNU (CMC) and vincristine, adriamycin and procarbazine (VAP). CAPO (Brower *et al.*, 1983) represents combination chemotherapy with cyclophosphamide, adriamycin, VP-16 and vincristine. VP/plat represents treatment with VP-16 and *cis*-platin which has been shown to be effective in the treatment of small cell lung cancer (Sierocki *et al.*, 1979) and HD MTX indicates treatment with high dose methotrexate as a single agent. The patient's response to this treatment is shown in the next column, with CR representing a complete response to therapy, PR a partial response and NR no response. Regimes and responses in parenthesis represent the effect of treatment in patients subsequently exposed to cytotoxic drugs, in whom the cell line was established prior to exposure to drugs. U/T: Untreated; U/K: Unknown. Cell line details previously described in Carney *et al.* (1985) and Gazdar *et al.* (1985).

(Carmichael et al., 1987a). To each well 20 $\mu$l of 10 × drug in PBS was added, with PBS added to control wells. Cells were incubated with or without drug for 4 days, allowing sufficient time for cell replication, drug induced cell death, and loss of enzymic activity, which generates the formazan product from the MTT substrate (Mossman, 1983). Following a 4 day incubation, 100 $\mu$g MTT (50 $\mu$l 2 mg ml$^{-1}$ solution was added to each well, and the plates incubated for 4 h. The microtitre plates were centrifuged at 450 g for 10 min, 220 $\mu$l supernatant removed using a Costar-96 Transpipette system, leaving ~30 $\mu$l residual medium in each well. MTT formazan crystals were then resolubilized by adding 150 $\mu$l 100% dimethylsulfoxide (DMSO) to each well. Plates were then agitated on a plate shaker for 5 min, following which spectrophotometric absorbance at 540 nm was immediately determined using a scanning multiwell spectrophotometer (Biotek Instruments Inc., Burlington, Vermont).

### *Drugs*

Drugs were obtained as formulated for clinical use, except for melphalan and BCNU, which were obtained from Sigma Chemical Co., St Louis, MO. All drugs were prepared freshly for each experiment at 100 × the maximal final concentration used. Melphalan was dissolved in acid ethanol and BCNU was dissolved in 50% ethanol, while the remaining drugs were dissolved in normal saline or water. All drugs were subsequently diluted using PBS and finally passed through a 0.45 $\mu$m sterile filter (Millipore Corporation, Bedford, MA) prior to use.

### *Statistical methods and study design*

Datum points in Figures 1 and 2 represent the mean of octuplicate tests at each drug concentration ± s.d. Data in Tables II and III represent the mean $IC_{50}$ ± s.d. of up to 3 experiments. For certain cell lines there was only one satisfactory experiment and the $IC_{50}$ is given without a standard deviation. Tables IV and V were done using the Spearman rank order analysis, taking into account Bonferroni criteria for the assessment of significance of multiple tests. Variation between histological sub-groups of lung cancer for each drug was analysed using the Kruskal–Wallis one way analysis of variance (Table VI) and individual groups were tested using the Mann–Whitney test (Table VII). All 30 cell lines were tested simultaneously, and the experiment repeated 3 times. Controls included media alone, media plus drug and an untreated cellular control. Each drug was tested at 10 drug concentrations with each concentration point representative of 8 replicate wells for each cell line. Graphs were prepared manually, with response curves generated using a best fit of the data. Response curves were plotted for all drugs, with the $IC_{50}$ values for each cell line determined graphically as the dose of drug causing a 50% reduction in absorbance compared with control values. Statistical calculations were performed using microcomputer programs (Statworks and Cricketgraph, Cricket Software, Philadelphia, PA).

### Results

### *Lung cancer cell lines and plating densities*

Table I lists the characteristics of the 30 lung cancer cell lines used in this study including histologic type, prior therapy, and response to therapy. The small cell lines nearly all grow as floating aggregates, while the non-small cell lung cancers grow as attached monolayers. In addition to their morphologic and biochemical differences (Carney et al., 1985;

**Figure 1** Cell survival 4 days after plating and drug exposure measured by the MTT assay for adriamycin, BCNU, *cis*-platin and melphalan for 3 different lung cancer cell lines. NCI-H209, untreated small cell lung, (■): NCI-H146, treated small cell lung cancer, (□): NCI-H322 non-small cell lung cancer, (▲). The datum points shown on each figure represent the mean ± s.d. for the 8 replicate determinations normalized to the control value which was set at 100%.

**Figure 2** Cell survival 4 days after plating and drug exposure measured by the MTT assay for VP-16, vinblastine and vincristine for 3 lung cancer cell lines. Cell lines and symbols are the same as for **Figure 1**. Datum points are the mean ±s.d. for the 8 replicate determinations normalized to the control value set at 100%.

Gazdar *et al.*, 1985), the lung cancer cell lines vary widely in their growth rates and cloning efficiencies. Thus, preliminary experiments were done to determine the optimal seeding density required for each cell line to be in the log phase of growth when the assay was terminated 4 days after plating (Table I). The mean absorbance for the control (untreated) wells for all 30 cell lines in the assays reported was 0.35, with a standard deviation of 0.12 between the different cell lines. For any one cell line, the standard deviation of control absorbance values among the 8 replicates was <20%.

*Effect of drugs on killing lung cancer cells in the MTT assay*

Tumour cell killing as a function of drug concentration was determined for the 7 drugs, using all 30 cell lines, and this was repeated in 3 experiments. The drugs were tested over the following concentration ranges: adriamycin 1–500 nM; BCNU 5–1000 μM; *cis*-platin 0.1–30 μM; melphalan 0.01–500 μM; vinblastine 0.1–100 nM; vincristine 0.1–100 nM and VP-16 0.1–100 μM. These concentration ranges gave 50% or greater reduction in the production of formazan product for the 30 cell lines allowing the determination of an $IC_{50}$ for each drug with each cell line. However, 2 cell lines, NCI-H125 and NCI-H157, were highly resistant to vinblastine, and determination of an $IC_{50}$ was not possible. One example from each of the 3 main treatment/histologic types (untreated SCLC, treated SCLC and non-SCLC) for each of the 7 drugs used are shown in Figures 1 and 2. The slopes of the dose-response curves varied considerably with the drugs used, as shown in Figures 1 and 2. Steep dose-response curves are seen with *cis*-platin and BCNU, whereas in contrast, shallow dose response curves are observed with VP-16 and vinblastine. Table II (for small cell lines) and Table III (for non-small cell lines) summarize the $IC_{50}$ data for all 30 cell lines.

*Reproducibility of the chemosensitivity profile of the lung cancer cell line panel*

When repeated dose-response curves were generated for cell lines within each experiment, the $IC_{50}$ values varied by approximately 15%. However, wider variations in $IC_{50}$ values were seen between experiments. We noted that the differences between experiments affected all of the cell lines tested for a particular drug and presumably represent factors we do not currently know how to control. However, the large standard deviations may be explained, in part, by the wide dosage range covered by each drug in this study. It was important to determine if the relative degree of chemosensitivity or resistance among the 30 cell lines for each drug was maintained between experiments. Thus, for each drug tested, we asked did the tumour cell lines retain their rank order of chemosensitivity relative to other members of the lung cancer cell line panel between experiments? To do this we performed a rank order analysis of the $IC_{50}$s for each drug as determined in separate experiments (Table IV). This analysis shows a highly significant correlation between experiments for the rank order of chemosensitivity of the tumour cell lines for all 7 drugs.

*Correlation of chemosensitivity between drugs for the lung cancer panel*

We then asked if rank order of sensitivity to one drug predicted rank order of sensitivity to other drugs. As is shown in Table V, rank order of sensitivity to certain drugs was highly correlated with sensitivity to many of the agents tested. Whether this is a manifestation of the multidrug resistance phenotype (Ling *et al.*, 1983) remains to be determined. Of interest, vinblastine sensitivity was only significantly correlated with vincristine sensitivity, while *cis*-platin and VP-16 rank order of chemosensitivities were not significantly correlated (Table V).

*Comparisons of chemosensitivity among different lung cancer groups*

We then asked if relative rank order of chemosensitivity to each drug was associated with the major clinical status groups, namely untreated small cell lung cancer, relapsed small cell lung cancer and non-small cell lung cancer. First, the Kruskal–Wallis one way analysis of variance was used to analyze the data from all 30 cell lines for each drug, to assess whether any significant differences between histologic or treatment groups could be detected (Table VI). This test is a non-parametric analysis based on the ranks of the dependant variable ($IC_{50}$) for the 3 lung cancer treatment/histologic classes), compared to the independent variable (drug). Highly significant differences in chemosensitivity were observed between lung cancer sub-groups for adriamycin, melphalan, vincristine and VP-16, while no significant differences were detected for BCNU, *cis*-platin and vinblastine. We then performed Mann–Whitney comparisons of untreated SCLC *vs.* treated SCLC and *vs.* non-SCLC, and compared treated SCLC *vs.* non-SCLC for rank order of sensitivity to each drug (Table VII). In all cases the signifi-

**Table II** Mean $IC_{50}$ values of small cell lung cancer lines ±s.d. determined from the survival curves of 3 replicate experiments

| Cell line | (nM) Adriamycin | (μM) BCNU | (μM) Cis-platin | (μM) Melphalan | (nM) Vinblastine | (nM) Vincristine | (μM) VP-16 |
|---|---|---|---|---|---|---|---|
| *Untreated Classic* | | | | | | | |
| NCI-H187 | 25.5 (22) | 35.8 (32) | 1.0 (0.5) | 1.8 (2) | 4.0 (2) | 0.8 (0.6) | 0.6 (0.5) |
| NCI-H209 | 24.8 (7.2) | 35.8 (19) | 0.3 (0.3) | 0.2 (0.1) | 3.3 (3) | 0.3 (0.1) | 0.5 (0.2) |
| NCI-H678 | 61.0 | NE | 3.4 (2.3) | 6.3 (8) | NE | NE | 0.40 |
| NCI-H719 | 10.1 (7.7) | 128 (56) | 3.8 (1.1) | 0.1 | 2.8 | NE | 0.31 (0.2) |
| NCI-H889 | 13.4 (6.5) | NE | 1.7 (1.6) | 3.6 (2) | 1.75 | 1.6 (0.8) | 0.6 (0.6) |
| *Untreated Variant* | | | | | | | |
| NCI-H417 | 12.9 (3) | 74.6 (16) | 2.0 (1.3) | 0.8 | 8.1 (4.7) | 2.2 (1.4) | 3.7 (2) |
| NCI-H526 | 37.0 (17) | 30.8 (25) | 0.7 (0.4) | 0.1 (0) | 3.7 (2.7) | 0.8 (0.7) | 1.2 (1) |
| *Treated Classic* | | | | | | | |
| NCI-H60 | 171 (134) | 36.0 (24) | 1.4 (0.3) | 2.8 (2.9) | 4.8 (5.4) | 1.4 (1.4) | 5.5 (3.8) |
| NCI-H69 | 127 (46) | 103 (51) | 2.0 (1) | 10.1 (4) | 5.0 (4.0) | 2.4 (0.7) | 18.3 (10) |
| NCI-H128 | 110 (126) | 78.5 (53) | 4.8 (0) | 20.8 (8) | 4.4 (2.8) | 4.9 (3.7) | 25.7 (27) |
| NCI-H146 | 144 (86) | 95 (13) | 3.7 (1) | 13.8 (8) | 10.6 (0) | 3.9 (1.6) | 2.9 (3) |
| NCI-H249 | 127 (123) | 75.9 (21) | 1.5 (0) | 10.2 (11) | 3.6 (3.3) | 1.0 (0.2) | 4.0 (3) |
| *Treated Variant* | | | | | | | |
| NCI-H82 | 94.0 (71) | 104 (51) | 1.1 (0) | 7.9 (6.2) | 3.4 (1.9) | 1.7 (0.4) | 10.5 (14) |
| NCI-H524 | 15.8 (3.9) | 43.6 (13) | 0.4 (0) | 2.8 (2.4) | 1.8 (1.7) | 1.0 (0.5) | 1.4 (0.3) |
| NCI-H841 | 231.0 (162) | 162 (29) | 21.0 (8.8) | 84.0 (23) | 5.9 (5) | 7.2 (5) | 9.7 (6.1) |

Mean $IC_{50}$ values for 7 drugs using 15 small cell lung cancer cell lines. $IC_{50}$ values are shown with the standard deviation in brackets. Where no bracket is shown, the $IC_{50}$ value is representative of one experiment only and NE represents no evaluable experiments.

**Table III** Mean $IC_{50}$ values of non-small cell lung cancer cell lines ±s.d. determined from the survival curve of 3 replicate experiments

| Cell line | (nM) Adriamycin | (μM) BCNU | (μM) Cis-platin | (μM) Melphalan | (nM) Vinblastine | (nM) Vincristine | (μM) VP-16 |
|---|---|---|---|---|---|---|---|
| *Adenocarcinoma* | | | | | | | |
| NCI-H23 | 38.7 (26) | 112.0 | 2.5 (2.5) | 8.7 (7) | 5.2 | 2.2 (1.3) | 1.3 |
| NCI-H125 | 216.0 (58) | 96.4 (52) | 1.5 (0.7) | 8.2 (3) | >100 | 1.9 (1.3) | 5.9 (3.7) |
| NCI-H358 | 85.0 (56) | 76.3 (9) | 5.9 (1.7) | 31.0 (9) | 16.0 (14) | 8.1 (2.6) | 10.3 (9) |
| NCI-H522 | 197.0 (72) | NE | 3.2 (2.0) | 30.3 (18) | 3.1 (3.0) | 1.2 (0.6) | 11.3 (1) |
| A549 | 57.7 (32) | 97.3 (6) | 1.5 (1.3) | 11.4 (7) | 4.4 (4.6) | 13.7 (11) | 1.9 (0.6) |
| *Squamous* | | | | | | | |
| NCI-H226 | 221 (90) | 162 (30) | 2.9 (2.0) | 56.3 (17) | 19.0 (10) | 22.9 (24) | 5.2 (0.5) |
| NCI-H520 | 411 (132) | 117 (20) | 3.4 (1.9) | 23.3 (9) | 3.2 (1.7) | 5.9 (3.6) | 40.9 (22) |
| *Adenosquamous* | | | | | | | |
| NCI-H596 | 813 (207) | 221 (15) | 9.1 (13) | 60.7 (30) | 20.4 (13) | 16.8 (20) | 79.0 (15) |
| NCI-H647 | 115 (10) | 118 (112) | 10.4 (10) | 55.3 (32) | 3.2 (1.8) | 6.5 (3.3) | 7.3 (7) |
| NCI-H322 | 173 (165) | 206 (123) | 10.2 (4) | 75.5 (51) | 3.8 (3.9) | 4.9 (2.2) | 23.9 (30) |
| *Large Cell Carcinoma* | | | | | | | |
| NCI-H157 | 238 (167) | 151 (55) | 16.8 (3) | 104.8 (60) | >100 | 115.0 (53) | 30.5 (13) |
| NCI-H460 | 16.5 (8) | 45.2 (10) | 1.7 (2) | 1.6 (0.4) | 6.2 | 3.5 (3.2) | 0.5 (1.1) |
| NCI-H661 | 130 (57) | 102.8 (42) | 4.4 (3) | 20.4 (19) | 1.8 (0.6) | 1.5 (1.2) | 1.0 |
| *Mesothelioma* | | | | | | | |
| NCI-H290 | 26.8 (14) | 51.7 (8) | 1.0 (0.4) | 3.7 (0.6) | 3.5 (3.4) | 2.3 (2.1) | 3.0 (0.1) |
| JMN | 40.2 (13) | 36.8 (10) | 2.3 (2.3) | 3.3 (1) | 9.2 (3.7) | 11.4 (8.2) | 1.7 (0.2) |

Mean $IC_{50}$ values for 7 drugs using 15 non-small cell lines. $IC_{50}$ values are shown with the standard deviation in brackets. Where no bracket is shown, the $IC_{50}$ value is representative of one experiment only and NE represents no evaluable experiments.

cant differences discovered by the Kruskal–Wallis test resulted from the lower $IC_{50}$ values for the untreated SCLC and their consequent ranking as the most sensitive cells. In addition, a significant difference for vincristine was found between treated SCLC and non-SCLC (Table VII).

### Discussion

In our previous work, we have shown the MTT assay to correlate extremely well with clonogenic and dye exclusion assays in generating response curves for both cytotoxic drugs and radiation (Carmichael *et al.*, 1987*a*,*b*). The drugs tested in these studies included adriamycin, *cis*-platin, melphalan, vincristine and VP-16. The current study applied this semi-automated assay to a detailed study of a much larger panel of 30 lung cancer cell lines, using 7 drugs (or their close relatives) commonly used in the treatment of lung cancer. The results show that:

1. Based on their relative $IC_{50}$ values, a reproducible rank order for chemosensitivity could be generated in the 30 line panel for each of the 7 drugs.
2. Small cell lung cancer lines derived from untreated patients had a significantly different rank order of sensitivity (were the most sensitive) to adriamycin, melphalan, vincristine and VP-16 compared to treated SCLC and non-SCLC, and were significantly more sensitive to BCNU and *cis*-platin when compared to non-SCLC.
3. The only significant difference between treated SCLC and non-SCLC was toward the drug vincristine.
4. No significant differences were observed between the 3 treatment/histologic groups for vinblastine.

**Table IV** Spearman rank order analysis of relative drug sensitivity of lung cancer cell lines

| *Drug* | *No. of paired observations* | *Spearman correlation* | *Significance (P value)* |
|---|---|---|---|
| Adriamycin | 16 | 0.923 | 0.0000003 |
| BCNU | 18 | 0.676 | 0.002 |
| *Cis*-platin | 11 | 0.884 | 0.0003 |
| Melphalan | 12 | 0.914 | 0.00003 |
| Vinblastine | 11 | 0.752 | 0.0076* |
| Vincristine | 13 | 0.874 | 0.00001 |
| VP-16 | 11 | 0.818 | 0.002 |

In independent experiments performed over a period of 3 months the $IC_{50}$s of the tumour cell lines were redetermined and the rank order of tumour cell sensitivity recalculated. Spearman correlation values (rho) and their significance were determined using the Statworks microcomputer program. Vinblastine: In repeat experiments Spearman correlations of 0.715 and 0.854 with significance values of $P = 0.0013$ and 0.0008 respectively were obtained indicating significant correlation of the rank order of chemosensitivity for this drug.

*Note that a $P$ value of $<0.002$ for 21 individual tests is equivalent to $P<0.05$ for a single test by Bonferroni criteria.

**Table V** Spearman rank order analysis of relative drug sensitivity of lung cancer cell lines to different drugs

| | *All cell lines* | |
|---|---|---|
| *Drugs* | *Specimen correlation (rho)* | *Significance (P value)* |
| Adriamycin *vs.* BCNU | 0.619 | 0.001 |
| Adriamycin *vs.* *cis*-platin | 0.529 | 0.003[a] |
| Adriamysin *vs.* melphalan | 0.813 | $4.55 \times 10^{-8}$ |
| Adriamysin *vs.* vinblastine | 0.383 | 0.04[a] |
| Adriamycin *vs.* vincristine | 0.475 | 0.011[a] |
| Adriamycin *vs.* VP-16 | 0.757 | $1.26 \times 10^{-6}$ |
| BCNU *vs.* *cis*-platin | 0.763 | $3.57 \times 10^{-6}$ |
| BCNU *vs.* melphalan | 0.750 | $6.66 \times 10^{-6}$ |
| BCNU *vs.* vinblastine | 0.165 | 0.41 |
| BCNU *vs.* vincristine | 0.658 | 0.00026 |
| BCNU *vs.* VP-16 | 0.561 | 0.0023 |
| *Cis*-platin *vs.* melphalan | 0.754 | $1.47 \times 10^{-6}$ |
| *Cis*-platin *vs.* vinblastine | 0.266 | 0.163[a] |
| *Cis*-platin *vs.* vincristine | 0.708 | $2.45 \times 10^{-5}$ |
| *Cis*-platin *vs.* VP-16 | 0.459 | 0.01 |
| Melphalan *vs.* vinblastine | 0.288 | 0.13[a] |
| Melphalan *vs.* vincristine | 0.688 | $5.05 \times 10^{-5}$ |
| Melphalan *vs.* VP-16 | 0.738 | $3.24 \times 10^{-6}$ |
| Vinblastine *vs.* vincristine | 0.581 | 0.001 |
| Vinblastine *vs.* VP-16 | 0.324 | 0.086[a] |
| Vincristine *vs.* VP-16 | 0.522 | 0.0044[a] |

[a]Note that a $P$ value of $<0.002$ for 21 individual tests is equivalent to $P<0.005$ for a single test by Bonferroni criteria.

**Table VI** Kruskal–Wallis one way analysis of variance comparing three major subgroups of lung cancer

| | *H-statistic* | *Significance (P value)* |
|---|---|---|
| Adriamycin | 11.029 | 0.004 |
| BCNU | 5.37 | 0.068 |
| *Cis*-platin | 4.031 | 0.133 |
| Melphalan | 12.804 | 0.002 |
| Vinblastine | 2.893 | 0.235 |
| Vincristine | 10.411 | 0.005 |
| VP-16 | 17.193 | 0.004 |

5. There was significant correlation of sensitivity and resistance between certain drugs (particularly adriamycin, melphalan and vincristine).
6. Vinblastine sensitivity was correlated only with vincristine sensitivity.
7. Sensitivity to *cis*-platin and VP-16 were not correlated.

Variation was observed between experiments, although the cause of this remains uncertain. A number of factors may have contributed including the wide dose ranges of drugs used in these studies. Also, for some drugs, the dose response curves were extremely shallow, making precise definition of the $IC_{50}$ difficult. Despite variation of the $IC_{50}$ values to different drugs of up to one log for the same cell line assayed at different times, we found the relative rank order of sensitivities of the 30 cell lines for any one drug to be highly correlated between assays. In view of this variation, it will be essential to include previously tested lung cancer cell lines of varying sensitivities to serve as reference points for subsequent assays, particularly for the testing of new anti-cancer drugs.

The MTT assay offers many advantages, as it is performed in microtitre plates with the production of a coloured formazan product. The simplicity of the assay permits the testing of multiple replicates and conditions with recording of results using commercially available ELISA plate readers. In fact, this paper summarizes data from over 40,000 test points. Other advantages of the assay include the low intratest variation between data points ($\pm 15\%$ s.d.) and the ability to test tumour cell lines with low cloning efficiencies or cell lines from which it may be difficult to generate single cell suspensions (Bertoncello *et al.*, 1982; Selby *et al.*, 1983). Currently, the limiting feature of this assay is data analysis that obviously requires the use of appropriate computer software, which is now becoming generally available.

Testing of primary tumour samples using both clonogenic (Hamburger & Salmon, 1977; Salmon *et al.*, 1980; Van Hoff *et al.*, 1981) and other assays (Weisenthal *et al.*, 1983; Bird *et al.*, 1985; Van Hoff *et al.*, 1985) has been shown to be a good predictor of the *in vivo* response in individual patients. The MTT assay, as described, would be unable to accomplish this as non-tumour cells also reduce the substrate. The assay requires a population of tumour cells free of normal cells and would fail to detect killing effects that were only expressed in a small sub-population of cells (such as effects only registered in clonogenic cells). We know that both human and Chinese hamster lung fibroblast cells reduce MTT. However, there is no further documented evidence for normal tissues reducing MTT. However, the use of tumour cell lines provides pure populations of tumour cells, and our previous work has shown a good correlation between results of the MTT and the clonogenic assays for the drugs tested in this report. Other workers using smaller numbers of cell lines and different assays have also concluded that there is a correlation between the clinical response of certain tumour types and the *in vitro* response using established cell lines of similar histology (Hill, 1983; Cole, 1986). In contrast to our finding that untreated SCLC lines were more sensitive than previously treated SCLC lines, Hug *et al.* (1984) found no differences in the chemosensitivity patterns of treated versus untreated breast cancer lines. The possibility exists that some of the differences observed in this study could be due to morphological differences, i.e., floating *versus* monolayer cells. However, this difference is unlikely to be of significance as a significant difference was observed between previously treated and untreated small cell lung cancer cells, virtually all of which grew as floating aggregates. Likewise, differences in growth rate were unlikely to be of significance, as the untreated small cell lines which had the longest doubling time were in fact the most sensitive.

Many of these findings could have potential clinical

**Table VII** Mann–Whitney rank order analysis of the three different classes of lung cancer lines

| *Drug tested* | *Untreated SCLC vs. treated SCLC* | *Untreated SCLC vs. non-SCLC* | *Treated SCLC vs. non-SCLC* |
|---|---|---|---|
| | | *P* value (one tailed)[a] | |
| Adriamycin | <0.005 | <0.005 | Not significant |
| BCNU | Not significant | <0.025 | Not significant |
| *Cis*-platin | Not significant | <0.05 | Not significant |
| Melphalan | <0.005 | <0.005 | Not significant |
| Vinblastine | Not significant | Not significant | Not significant |
| Vincristine | <0.05 | <0.005 | <0.05 |
| VP-16 | <0.005 | <0.005 | Not significant |

[a]The rank order of all 3 major classes were compared. The question was asked whether the untreated SCLC lines had lower $IC_{50}$ ratings for each drug, while the same question was asked for the treated SCLC *vs.* the non-SCLC lines. In two tailed tests, all of the comparisons significant at the <0.005 level in one tailed tests are significant at <0.01, those significant at the <0.025 level are significant at the <0.05 level, while those significant at the <0.05 level have a *P* value of <0.1. As suggested by Cricket software, statistical tables (Goldstein, 1964) were used to determine the *P* values for the Mann–Whitney U statistic generated.

applications or corollaries. In these studies we have concentrated on drugs actually used in clinical treatment, whenever possible, such as adriamycin, *cis*-platin, vincristine and VP-16, or similar classes of drug to those more frequently used such as BCNU and melphalan. It was of interest to see that *cis*-platin and VP-16 sensitivity patterns were different, and that the 3 lung cancer treatment groups did not differ significantly with respect to *cis*-platin sensitivity. The combination of *cis*-platin and a vinca alkaloid appears to be active against both small cell (Hug *et al.*, 1984) and non-small cell lung cancer (Gralla *et al.*, 1981). In addition, only one of the treated small cell lines (NCI-H841) came from a patient whose tumour had relapsed on *cis*-platin and VP-16 combination chemotherapy, and this line had the highest $IC_{50}$ value for *cis*-platin.

There are many potential ways for analysing the data obtained from these studies. Different inhibitory concentration points such as the $IC_{50}$, $IC_{70}$ or $IC_{90}$ could be used, or area under the curve of drug concentrations, as well as utilizing different incubation times. Our major findings are that a panel of lung cancer cell lines can be rank ordered as to relative chemosensitivity and that this correlates with histologic type and prior therapy status. While we have emphasized the three major groups, each contains subgroups as well, such as classic and variant SCLC, and several non-SCLC histologic subtypes. In these groups there are exceptions with a treated SCLC line (NCI-H524) and a non-SCLC line (NCI-H460) both sensitive to many drugs. However, clinical experience shows that the occasional relapsed SCLC or non-SCLC patient proves to be sensitive to chemotherapy, while the occasional untreated SCLC can be resistant to therapy. Prospective clinical trials which we are now conducting are aimed at clarifying the relationship between *in vitro* and clinical responses in individual patients. Nevertheless, the results reported here should support and encourage the use of the combination of the semi-automated MTT assay and the panel of lung cancer cell lines to begin clinical *in vitro* correlations of drug sensitivity and resistance.

### References

BERTONCELLO, I., BRADLEY, T.R., CAMPBELL, J.J. & 6 others (1982). Limitations of the clonal agar assay for the assessment of primary human ovarian tumour biopsies. *Br. J. Cancer*, **45**, 803.

BIRD, M.C., BOSANQUET, A.G. & GILBEY, E.D. (1985). *In vitro* determination of tumour chemosensitivity in haematological malignancies. *Haematol. Oncol.*, **3**, 1.

BLACK, M.M. & SPEER, F.D. (1954). Further observations on the effects of cancer chemotherapeutic agents on the *in vitro* dehydrogenase activity of cancer tissue. *J. Natl Cancer Inst.*, **14**, 1147.

BROWER, M., IHDE, D.C., JOHNSTON-EARLY, A. & 7 others (1983). Treatment of extensive stage small cell bronchogenic carcinoma. Effects of variation in intensity of induction chemotherapy. *Amer. J. Med.*, 993.

CARMICHAEL, J., DEGRAFF, W.G., GAZDAR, A.F., MINNA, J.D. & MITCHELL, J.B. (1987*a*). Evaluation of a tetrazolium based semi-automated colorimetric assay. I: Assessment of chemosensitivity testing. *Cancer Res.*, **47**, 936.

CARMICHAEL, J., DEGRAFF, W.G., GAZDAR, A.F., MINNA, J.D. & MITCHELL, J.B. (1987*b*). Evaluation of tetrazolium based semi-automated colorimetric assay. II: Assessment of radiosensitivity. *Cancer Res.*, **47**, 943.

CARNEY, D.N., GAZDAR, A.F., BEPLER, G. & 5 others (1985). Establishment and identification of small cell lung cancer cell lines having classic and variant features. *Cancer Res.*, **45**, 2913.

COHEN, M.H., IHDE, D.C., BUNN, P.A. Jr. & 6 others (1979). Cycle alternating combination chemotherapy for small cell bronchogenic carcinoma. *Cancer Treat. Rep.*, **63**, 163.

COLE, S.P.C. (1986). Rapid chemosensitivity testing of human lung tumour cells using the MTT assay. *Cancer Chemother. Pharmacol.*, **17**, 259.

FRIEDMAN, O.M., MYLES, A. & COLVIN, M. (1979). Cyclophosphamide and related phosphoramide mustards: current status and future prospects. *Adv. Cancer Chemotherapy*, **1**, 159.

GAZDAR, A.F., CARNEY, D.N., NAU, M.N. & MINNA, J.D. (1985). Characterization of variant subclasses of cell lines derived from small cell lung cancer having distinctive biochemical, morphological, and growth properties. *Cancer Res.*, **45**, 2924.

GOLDSTEIN, A. (1964). *Biostatistics: An Introductory Text.* p. 257. Macmillan Co.: New York.

GRALLA, R.J., CASPER, E.S., KELSEN, D.P. & 5 others (1981). *Cis*-platin and vindesine combination chemotherapy for advanced carcinoma of the lung: A randomized trial investigating two dosage schedules. *Ann Intern. Med.*, **95**, 414.

HAMBURGER, A.W. & SALMON, S.E. (1977). Primary bioassay of human tumour stem cells. *Science*, **197**, 461.

HILL, B.T. (1983). Use of continuous human tumour cell lines to evaluate drugs by clonogenic assays. In *Human Tumour Drug Sensitivity Testing In Vitro,* Dendy, P.P. & Hill, B.T. (eds) p. 129. Academic Press, London.

HUG, V., THAMES, H., HAYNES, M., BLUMENSCHEIN, G., DREWINKO, B. & SPITZER, G. (1984). Use of normalized drug sensitivities of human breast tumours for clinical correlations and for comparisons of drug activities. In *Human Tumour Cloning,* Salmon, S.E. & Trent, J.M. (eds) p. 563. Grune & Stratton.

IDHE, D.C. & BUNN, P.A. (1982). Chemotherapy of small cell bronchogenic carcinoma. In *Recent Advances in Clinical Oncology,* Williams, C.J. & Whitehouse, J.M.A. (eds) p. 305. Churchill-Livingstone: Edinburgh & London.

JOHNSON, B.E., IHDE, D.C., BUNN, P.A., *et al.* (1985). Patients with small cell lung cancer treated with combination chemotherapy, with or without irradiation. *Ann. Intern. Med.*, **103,** 430.

KLASTERSKY, J., LONGEVAL, E., NICAISE, C. & WEERTS, D. (1982). Etoposide and *cis*-platinum in non-small cell bronchogenic carcinoma. *Cancer Test Rev.*, **9,** 133.

KONDO, T. & OHKUBO, K. (1967). *In vitro* test for prediction of side effects of carcinostatic agents. *Gann*, **58,** 349.

LING, V., KARTNER, N., SUDO, T., SLIMINOVITCH, L. & RIORDAN, J.R. (1983). Multidrug-resistance phenotype in Chinese hamster ovary cells. *Cancer Treat. Rep.*, **67,** 869.

LIVINGSTON, R.B. (1977). Combination chemotherapy of bronchogenic carcinoma. I. Non-oat cell. *Cancer Treat. Rev.*, **4,** 153.

MOSSMAN, T. (1983). Rapid colorimetric assay for cellular growth and survival: application to proliferation and cytotoxicity assays. *J. Immunol. Meths.*, **65,** 55.

SALMON, S.E., ALBERTS, D.S., DURIE, B.G.M. & 5 others (1980). Clinical correlations of drug sensitivity in the human tumour stem cell assay. *Recent Results Cancer Res.*, **74,** 300.

SELBY, P., BUICK, R.N. & TANNOCK, I. (1983). A critical appraisal of the 'Human tumour stem cell assay'. *N. Engl. J. Med.*, **308,** 129.

SIEROCKI, J.S., HILARIS, B.S., HOPFAN, S. & 4 others (1979). *cis*-Dichlorodiammineplatinum(II) and VP-16-213: an active induction regimen for small cell carcinoma of the lung. *Cancer Treat. Rep.*, **63,** 1593.

VAN HOFF, D.D., CASPER, H., BRADLEY, E., SANDBACK, J., JONES, D. & MAKUCH, R. (1981). Association between human tumour colony-forming assay results and response of an individual patient's tumor to chemotherapy. *Amer. J. Med.*, **70,** 1027.

VAN HOFF, D.D., FORSETH, B. & WARFEL, L.E. (1985). Use of a radiometric system to screen for antineoplastic agents: Correlation with a human tumour cloning system. *Cancer Res.*, **45,** 4032.

WEISENTHAL, L.M., MORSDEN, J.A., DILL, P.L. & MACALUSO, C.K. (1983). A novel dye exclusion method for testing *in vitro* chemosensitivity of human tumours. *Cancer Res.*, **43,** 749.